# Pregnancy Following Metabolic–Bariatric Surgery in a Woman With Potential Premature Ovarian Failure: A Case Report

**DOI:** 10.1155/2024/4707627

**Published:** 2024-09-30

**Authors:** Raheleh Moradi, Maryam Kashanian, Somayeh Mokhber, Abdolreza Pazouki

**Affiliations:** ^1^ Minimally Invasive Surgery Research Center Iran University of Medical Sciences, Tehran, Iran; ^2^ Shahid Akbarabadi Clinical Research Development Unit (ShCRDU) Iran University of Medical Sciences (IUMS), Tehran, Iran; ^3^ Center of Excellence of International Federation for Surgery of Obesity Hazrat-e-Rasool Hospital, Tehran, Iran

**Keywords:** bariatric surgery, case reports, fertility, pregnancy, primary ovarian insufficiency

## Abstract

Premature ovarian insufficiency (POI) is associated with decreased ovulation in the precursor stage which leads to ovarian failure in the end stage. Metabolic–bariatric surgery (MBS) can improve women's reproductive status, including the release of sex hormones, ovulation, and fertilization. Here, we report a spontaneous pregnancy following MBS despite potential ovarian insufficiency. A 38-year-old woman with severe obesity underwent three cycles of assisted reproduction that were not successful. Oligomenorrhea ≥ 4 months, laboratory indices, and previous poor ovarian response approved the diagnosis of diminished ovarian reserve and could be considered as the precursor stage of POI. Then a gastric bypass was applied, and a spontaneous pregnancy occurred in the 22^nd^ month after surgery, with 45.80% reduction in body mass index. MBS in women with obesity and idiopathic ovarian insufficiency may increase the chance of spontaneous ovulation and successful pregnancy.

## 1. Introduction

The ongoing pandemic of obesity is expanding, and metabolic–bariatric surgery (MBS) may be the last effective way to induce significant weight loss. Eighty percent of bariatric patients are women, and half of these are at reproductive age [1]. Recent research revealed that MBS could improve women's reproductive status including release of sex hormones, ovulation, and fertilization [2]. In a randomized controlled trial which compared MBS and medical care for obesity and infertility related to polycystic ovary syndrome, Samarasinghe et al. [3] found that women in the surgical group had significantly more spontaneous ovulations compared with the medical group.

Although there is not uniformly accepted definition of diminished ovarian reserve (DOR), it is diagnosed by using the laboratory measurements such as increased basal follicle-stimulating hormone (FSH) levels or FSH/luteinizing hormone (LH) ratio, low anti-Müllerian hormone (AMH), and low antral follicle count, as well as Bologna criteria of poor ovarian response (POR) [4, 5].

Premature ovarian insufficiency (POI), with a prevalence range of 0.9% to 2%, is clinically characterized by oligo/amenorrhea, increased levels of gonadotropins, and hypoestrogenism [6]. This condition can be transient or progressive and usually leads to eventual premature menopause. According to FSH levels, POI has been subdivided into three consecutive but progressive stages: precursor POI (10 IU/L < FSH ≤ 25 IU/L), early POI (25 IU/L < FSH ≤ 40 IU/L), and the end stage of POI that is premature ovarian failure (FSH > 40 IU/L) [7]. In this report, we present a successful pregnancy following MBS in a case with DOR who was considered to be in the precursor stage of POI.

## 2. Case Presentation

**Figure 1:**
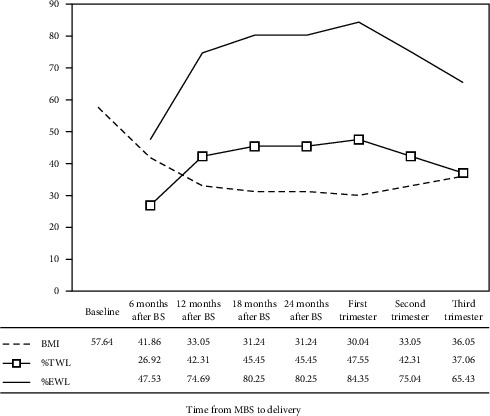
Trend of body mass index (BMI), percent total weight loss (%TWL), and percent excess weight loss (%EWL) from day of metabolic–bariatric surgery (MBS) to delivery.

A 38-year-old woman, nulligravida with a history of infertility for 9 years, was presented due to a spontaneous pregnancy, 22 months after MBS. Data were collected with the written informed consent of the client. A primary infertility was diagnosed because of the unilateral tubal factor and POR. Before MBS, the woman underwent three cycles of assisted reproduction including in vitro fertilization with intracytoplasmic sperm injection that were not successful. One-anastomosis gastric bypass was applied, when her body mass index (BMI) was 57.64 kg/m^2^. The trend of weight changes, from the day of surgery to delivery, is shown in Figure 1.

In the obstetrical history of patient, there were the irregular menstruation and left ovarian cystectomy. By hysterosalpingography, a left hydrosalpinx was confirmed. According to the literature, laboratory tests and the history of POR, related to an oocyte retrieval less than three with conventional stimulation protocol, approved a DOR diagnosis [4, 5]. Laboratory tests showed FSH at 10.11 (3.03–8.08 IU/L), LH at 8.06 mIU/mL (1.8–11.78 mIU/mL), estradiol (E2) at 52 (21–251 pg/mL), and AMH at 0.1 (2–6.8 ng/mL). Although DOR is different from POI, a history of oligomenorrhea ≥ 4 months, elevated FSH, and decreased AMH could consider as a precursor POI.

After MBS, contraception was recommended for 12 months, regarding the potential adverse effects of rapid weight loss on pregnancy outcome. In the 22^nd^ month after surgery, a spontaneous conception occurred, and then an intrauterine gestation of 9 weeks was identified by abdominal ultrasound. The mother was monitored for gestational weight gain, nutritional supplementation, and fetal health by a multidisciplinary team including the perinatologist, bariatric surgeon, and nutritionist. At a gestational age of 18 weeks, the mother underwent a cerclage procedure due to painless dilation of cervix with length of 23 mm. For an uncomplicated pregnancy without labor, pregnancy was terminated at 37 weeks to balance the risk of preterm birth against that of cervical laceration from a cerclage in place with labor contractions. A girl weighing 2960 g was born who was admitted to the neonatal intensive care unit, due to a mild respiratory distress. After 7 days of hospitalization, the baby was discharged healthy.

## 3. Discussion

In this report, a pregnancy following MBS was described in a woman with severe obesity and a history of infertility. Spontaneous conception occurred with a 45.80% loss in BMI, despite the elevated gonadotropins and declined AMH discussed as DOR and precursor POI. While previous studies reported spontaneous pregnancy following MBS or POI [8–10], in this case, pregnancy was detected after both MBS and POI.

A spontaneous conception was reported 6 months after diagnosis of premature ovarian failure and hormone replacement therapy [8]. The increase of gonadotropins, decrease of AMH, and hypoestrogenism were significant, compared to our case, indicating that ovarian insufficiency is progressive. Early diagnosis of POI is very important for starting hormone replacement therapy, delaying menopausal complications, and even in spontaneous or assisted fertility.

After 10 years of amenorrhea, a spontaneous pregnancy was diagnosed in a woman who was diagnosed with POI [9]. In this case, despite long-term amenorrhea and discontinuation of hormone replacement therapy, high levels of E2 and AMH were shown, compared to other cases. POI is characterized by intermittent ovarian function, while a physiological menopause is generally an irreversible event. Spontaneous conception has been observed in about 5% of women with POI, although its rate varies across the spectrum of pathological oocyte declining [8, 9]. Laboratory values of gonadotropins and E2 can be an important predictor of resumption of ovarian activity.

Two months after MBS, a pregnancy of 8 weeks was diagnosed in a 30-year-old woman with infertility for 11 years [10]. In the first trimester of pregnancy, BMI, percent total weight loss (%TWL), and excess weight loss (%EWL) were reported as 36 kg/m^2^, 24%, and 52%, respectively, which were comparable to the same values (30 kg/m^2^, 47%, and 84%) in the present case. Delaying pregnancy following MBS is associated with favorable weight loss outcomes. In addition, a positive trend in gestational weight gain, versus stopping or losing weight, leads to optimal maternal and perinatal outcomes.

In this case, contraception was recommended up to 12 months after surgery, because there was both rapid weight loss and an unstable nutritional status contrasted with gestational weight gain and nutritional requirements. Although there is an agreement to delay pregnancy after MBS, the determination of an optimal time is controversial [11, 12]. A shorter interval can be considered in cases of advanced maternal age or DOR, and the benefits of postponing pregnancy to achieve weight loss must be balanced against the risk of reduced fertility [13].

## 4. Conclusion

**Figure 2:**
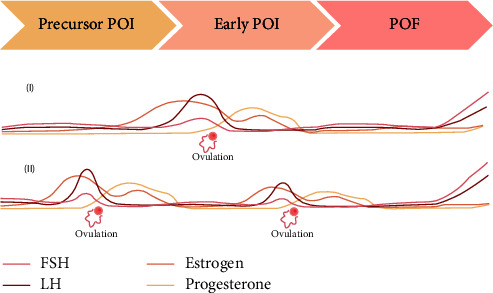
The spontaneous ovulation before (I) and after (II) metabolic–bariatric surgery (MBS). In primary stages of ovarian insufficiency, MBS in women with obesity can increase the chance of spontaneous ovulation and potential pregnancy by weight loss and metabolic correction without interrupting the progressive ovarian insufficiency.

In primary stages of ovarian insufficiency, MBS in women with obesity may increase the chance of spontaneous ovulation and potential pregnancy by weight loss and metabolic correction without interrupting the progressive ovarian insufficiency (Figure 2). In conclusion, this case report was presented to address future studies and personalize the clinical practice in women with obesity and a diagnosis of POI.

## Data Availability

Data are available from the corresponding author on reasonable request.
